# Supplementary material for: Private benefit of β-lactamase dictates selection dynamics of combination antibiotic treatment
Source: Nat Commun. 2024 Sep 27;15:8337. doi: 10.1038/s41467-024-52711-w (PMC11436977; doi:10.1038/s41467-024-52711-w)
Supplement: Supplementary file 1 — Supplementary Information [file 41467_2024_52711_MOESM1_ESM.pdf]

Supplementary information

1. Formulation of the base model

We model the dynamics of a bacterial system with resistant and sensitive subpopulations responding to a β-lactam/β-lactamase inhibitor combination as the interactions between five main components: sensitive population density (n_s), resistant population density (n_r), nutrient level (s), antibiotic concentration (a), and Bla concentration (b).

We expanded our ordinary differential equation model from Meredith et al.[18] to account for a sensitive subpopulation, costs (α) and benefits (β) of Bla production, antibiotic degradation by living resistant cells (φ), and the effect of Bla inhibitor (i) on Bla activity (d_b) and private benefit (modulated by c). The model specified in Meredith et al. also specifies parameters for nutrient recycling efficiency (ξ), antibiotic degradation by Bla (κ_b), basal antibiotic degradation (d_a). The effects of antibiotic and inhibitor are both saturating according to a Hill equation with Hill coefficients (h_a) and (h_i) respectively, which reflect the steepness of the dose-response curve.

Our model is as follows:

$$\frac{dn_s}{d\tau} = (g - l)n_s \quad \text{S1}$$

$$\frac{dn_r}{d\tau} = (\alpha g - \beta l)n_r \quad \text{S2}$$

$$\frac{ds}{d\tau} = (\xi l - g)n_s + (\xi\beta l - \alpha g)n_r \quad \text{S3}$$

$$\frac{da}{d\tau} = -\kappa_b ba - \varphi n_r a - d_a a \quad \text{S4}$$

$$\frac{db}{d\tau} = \beta l n_r - d_b \iota b \quad \text{S5}$$

$$g = \frac{s}{1+s} \quad \text{S6}$$

$$l = \gamma \frac{a^{h_a}}{1+a^{h_a}} g \quad \text{S7}$$

$$\iota = \frac{i^{h_i}}{1+i^{h_i}} \quad \text{S8}$$

$$\beta = \beta_{min} + c(1 - \beta_{min})\iota \quad \text{S9}$$

$$\varphi = \varphi_{max}(1 - c\iota) \quad \text{S10}$$

The base model, as in Meredith et al., makes several assumptions to formulate the dimensionless equations:

(1) Growth follows Monod growth kinetics, where nutrient level (s) is scaled with respect to the Monod constant. The maximum growth rate = 1/hr (when nutrient is saturating and there is no burden).

(2) The antibiotic concentration (a) is scaled with respect to IC50.

(3) The inhibitor concentration (i) is scaled with respect to the half-maximal inhibition concentration.

(4) The lysis rate (l) is proportional to the growth rate (g) with a maximum slope (γ) that increases with the antibiotic concentration (a). Though the linear correlation between l and g can be more complex[74,75], the additional complexity does not change the qualitative aspects of our conclusions.

We used initial conditions of $n_s(0) = 0.2$, $n_r(0) = 0.2$, $s(0) = 4$, $1 < a(0) < 100$, and $b(0) = 0$ for all simulations not involving the clinical isolates.

2. Parameters for the base model

We use parameter values consistent with our previous work in Meredith et al.[18] Unless otherwise specified, parameter values used were denoted as "Initial Value" below. When randomized parameter sets were generated to represent unique strains, parameters were uniformly randomized across the ranges given below.

Parameters	Initial Value	Randomized Range
α	0.95	0.75–1
β_{min}	0.9	0–1
ξ	0.8	0–1
d_a	0.02	-
κ_b	0.35	0–1
d_b	1	1–10
γ	1.38	1.1–1.4
h_a	3	1–5
i	0.1–10	-
h_i	2	1–5
φ_{max}	1	0–5
c	0.7	0–1

3. Criterion for Selective Response

The selective response of a mixed population to a combination treatment depends on the relative growth rates of the two populations. In general, the treatment enriches the resistant

population if the net growth rate of resistant cells, ρ_r, is greater than the net growth rate of sensitive cells, ρ_s. In other words, the treatment enriches the resistant population if:

$$\rho_r > \rho_s \qquad \text{S11}$$

$$\alpha g - \beta l > g - l \qquad \text{S12}$$

$$1 - \beta > \frac{1-\alpha}{l/g} \qquad \text{S13}$$

Note that S13 (also presented as Eq 1) is generally applicable regardless of the form of the lysis and growth terms.

To simplify our analysis, we consider the selection dynamics where antibiotic and inhibitor concentrations are saturating and before substantial antibiotic degradation has occurred. In this case, $l = \gamma g,\ \iota = 1,\ \beta = \beta_{min} + c(1 - \beta_{min})$. Accordingly, the criterion for enriching resistant cells becomes:

$$(1 - c)(1 - \beta_{min}) > \frac{1-\alpha}{\gamma},$$

which is presented as Eq. 2.

This simplified criterion allows the differentiation of strains for which combination treatment can select against resistance from strains for which it cannot, providing an analytical explanation for the importance of α, β_{min}, and c in determining the evolutionary response.

If horizontal gene transfer is present, the forms of the cell density equations in the model become:

$$\frac{dn_s}{d\tau} = (g - l)n_s - \eta n_r n_s + \sigma n_r \qquad \text{S14}$$

$$\frac{dn_r}{d\tau} = (\alpha g - \beta l)n_r + \eta n_r n_s - \sigma n_r \qquad \text{S15}$$

where η is the plasmid transfer rate and σ is the plasmid loss rate. As above, we note that treatment enriches the resistant population if its net growth rate, ρ_r, is greater than that of the sensitive cells, ρ_s. The relevant inequality becomes:

$$\alpha g - \beta l + \eta n_s - \sigma > g - l - \eta n_r + \sigma \frac{n_r}{n_s} \qquad \text{S16}$$

Using the assumptions specified above, we again note that in this case: $l = \gamma g,\ \iota = 1,\ \beta = \beta_{min} + c(1 - \beta_{min})$. Additionally, the initial values for the resistant (n_{r0}) and sensitive

(n_{s0}) population sizes, as well as the initial growth rate as a function of the initial nutrient ($\frac{s_0}{1+s_0}$), appear in the final form of the criterion. The criterion for enriching resistant cells thus becomes:

$$(1-c)(1-\beta_{min}) + \frac{\eta(n_{r0}+n_{s0})}{\gamma g_0} > \frac{1-\alpha}{\gamma} + \frac{\sigma(n_{r0}+n_{s0})}{n_{s0}\gamma g_0},$$

which is presented as Eq. 3.

4. Model formulation for estimating parameters from clinical isolates

The base model (Eqs S1–S10) was formulated to capture the qualitative features of the selection dynamics and was not intended to allow best fit to experimental growth curves. To allow reliable estimates of parameters directly from the experimental data collected on clinical isolates, we introduced several revisions to the base model. These revisions serve the sole purpose of improving the fitting to the experimental data; they do not change the qualitative predictions from the base model.

These data were collected from 311 clonal populations, each under three conditions: no drugs, with antibiotic, and with antibiotic and Bla inhibitor. For these data, the antibiotic and the Bla inhibitor were added in excess (see Methods).

Since these measurements were done on clonal populations, we only consider a single population in the revised model. We further revised the growth term, such that the effective growth rate is described by a more expressive function to better capture the complexity of experimental data. Here, growth is dependent on a carrying capacity N_m rather than on a nutrient concentration, which has been removed from the model.

$$\frac{dn}{dt} = (\mu - \beta l)\, n, \qquad \text{S16}$$

$$\mu = \left(\frac{\mu_{max}}{1+\left(\frac{n}{N_m K_s}\right)^{\theta}}\right)\left(1-\frac{n}{N_m}\right). \qquad \text{S17}$$

In addition, we account for the time-delayed lysis of a population by assuming that lysis can only occur if the population density exceeds L_n[75,76]. Based on experimental data, we also incorporate lysis from the inhibitor.

$$l = \gamma g \frac{a^{h_a} + \left(\frac{i}{K_i}\right)^{h_i}}{1 + a^{h_a} + \left(\frac{i}{K_i}\right)^{h_i}} \qquad \text{S18}$$

We assume that the turnover of the antibiotic follows Michaelis-Menten kinetics, with the Michaelis-Menten constant (K_a) being scaled with respect to the IC50 of the antibiotic.

$$\frac{da}{dt} = -(\kappa_b b + \phi n)\frac{a}{K_a + a} - d_a a, \qquad \text{S19}$$

Finally, we assume that there is small, basal-level turnover of free Bla enzyme.

$$\frac{db}{d\tau} = \beta l n_r - d_b i b - d_{b0} b \qquad \text{S20}$$

5. Parameter estimation

We used the Python Scipy.optimize.minimize function, using the Nelder-Mead algorithm, to estimate model parameters. Growth curves for each strain under each condition were collected in 12 replicates, and parameter estimates were done on the average of the 12 replicate growth curves. We conducted ten rounds of estimation. For each round, the initial guess for each parameter was chosen from a Gaussian distribution within the parameter bounds. The parameters were estimated in two steps.

In Step 1, we estimated the growth-related parameters (μ_{max}, K_s, and θ) in Eq S14, based solely on the growth curves in the absence of drug treatment. We found that these parameters can be estimated with high confidence and little variation in repeated attempts (Supplementary Figure S7).

In Step 2, we fixed these three parameters to the mean of their estimated values and conducted estimates for the seven remaining parameters using experimental data from all three conditions. For all parameters, bounds and mean and standard deviation for the distribution of initial guesses were as follows:

Parameters	Bounds	Gaussian mean	Gaussian S.D.
μ_{max}	0–4	2	0.67
K_s	0–0.4	0.2	0.067
θ	0–5	2.5	0.83
β_{min}	0–1	0.5	0.17
c	0–1	0.146	0.049
γ	0–6	1.35	0.18
L_n	0–0.8	0.4	0.13
κ_b	0–6	3	1
φ_{max}	0–6	3	1
d_b	0–6	3	1

Other parameters for basal level degradation, constants for antibiotic and inhibitor degradation, and carrying capacity were fixed at the following values: $d_a = 0.001$; $h_a = 3$; $h_a = 3$; $K_a = 1$; $K_i = 15$; $N_m = 3$. We expect these parameters to vary less between strains and, from previous modeling analysis (Supplementary Figure S3) affect the selection dynamics less.

Supplementary Table ST1. Keio strains included in the community coculture.

Keio ID	JW ID	Gene name
1	JW5419	*yfjP*
2	JW5438	*ygbF*
4	JW5481	*mltC*
5	JW5776	*sgcX*
7	JW0696	*ybfD*
9	JW2729	*ygcK*
11	JW1464	*narU*
12	JW5198	*yciX*
13	JW5422	*ypjA*
15	JW5456	*ygeI*
19	JW1455	*ydcC*
20	JW1822	*yebS*
27	JW5457	*pbl*
31	JW3451	*yhhI*
33	JW0746	*modA*
38	JW5444	*ygcE*
45	JW3381	*malT*
47	JW4367	*thrL*
52	JW5532	
54	JW0689	*rhsC*
55	JW0323	*prpB*
59	JW5064	
64	JW5577	*yigZ*
67	JW1730	*ydjQ*
69	JW1651	*ydhB*
70	JW3530	*glyS*
76	JW5646	*envC*
78	JW0486	*rhsD*
80	JW0820	*yliH*
88	JW5707	*gspD*
90	JW1451	*rhsE*
93	JW3650	*yidF*
96	JW2862	*xerD*

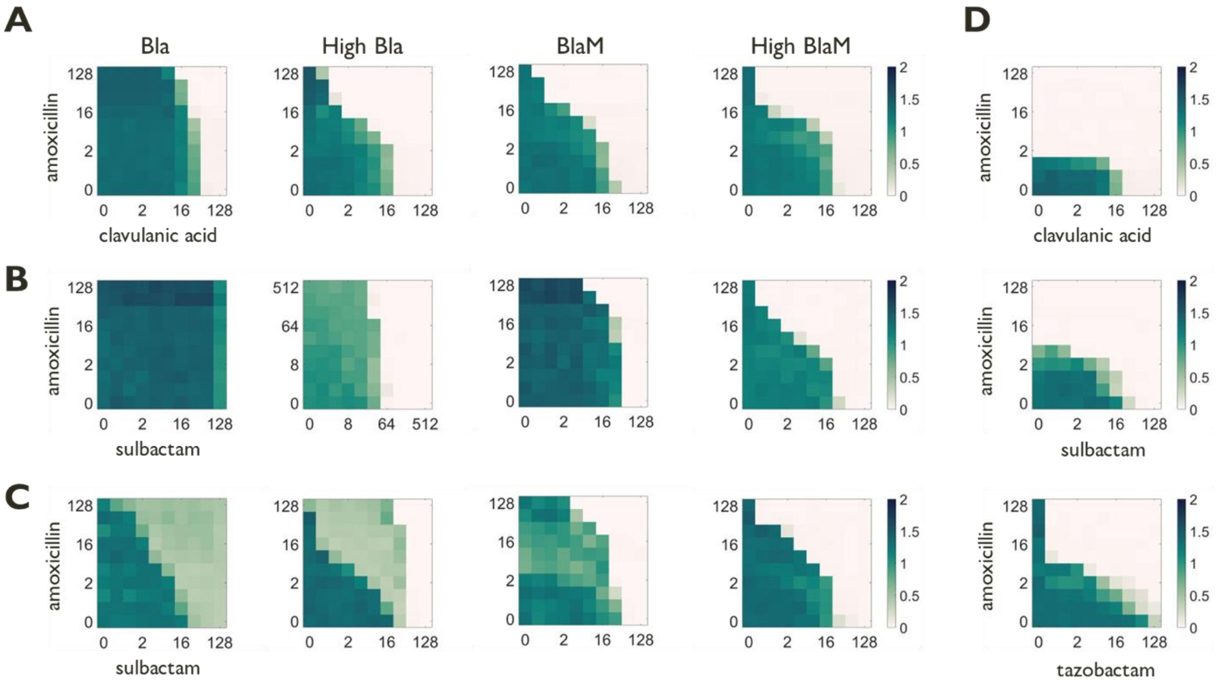

Supplementary Figure S1. Effects of Bla variant and copy number on effective doses and mixture results as seen in Figure 1 are consistent across different Bla inhibitors. High-throughput measurement of beta-lactam/beta-lactamase inhibitor dose response matrices. Color reports OD600 at 24 hours (*n*=3 biological replicates).

A. Dose response matrices for clonal strains treated with amoxicillin and clavulanic acid.
B. Dose response matrices for clonal strains treated with amoxicillin and sulbactam.
C. Dose response matrices for mixed populations of plasmid-free and plasmid-carrying strains treated with amoxicillin and sulbactam.
D. Dose response matrices for plasmid-free strains treated with amoxicillin and clavulanic acid (top), sulbactam (middle), and tazobactam (bottom).

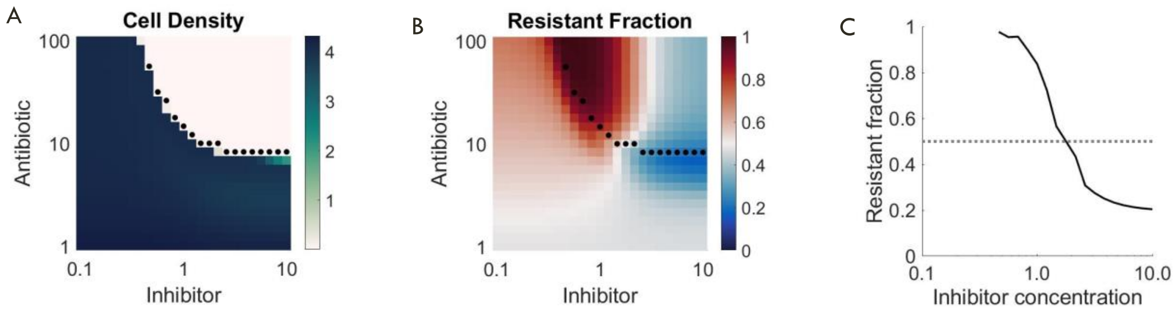

Supplementary Figure S2. Same cell density does not equal same resistant fraction. Black dots represent, for each inhibitor concentration, the lowest antibiotic concentration that leads to a final cell density below a threshold of 0.1 (A). These doses of equal effect, also known as an isobole, have very different final compositions (B). Final resistant fraction for doses along the minimally effective isobole are plotted by inhibitor concentration (C). Some doses lead to majority-resistant populations, while others lead to majority-sensitive populations.

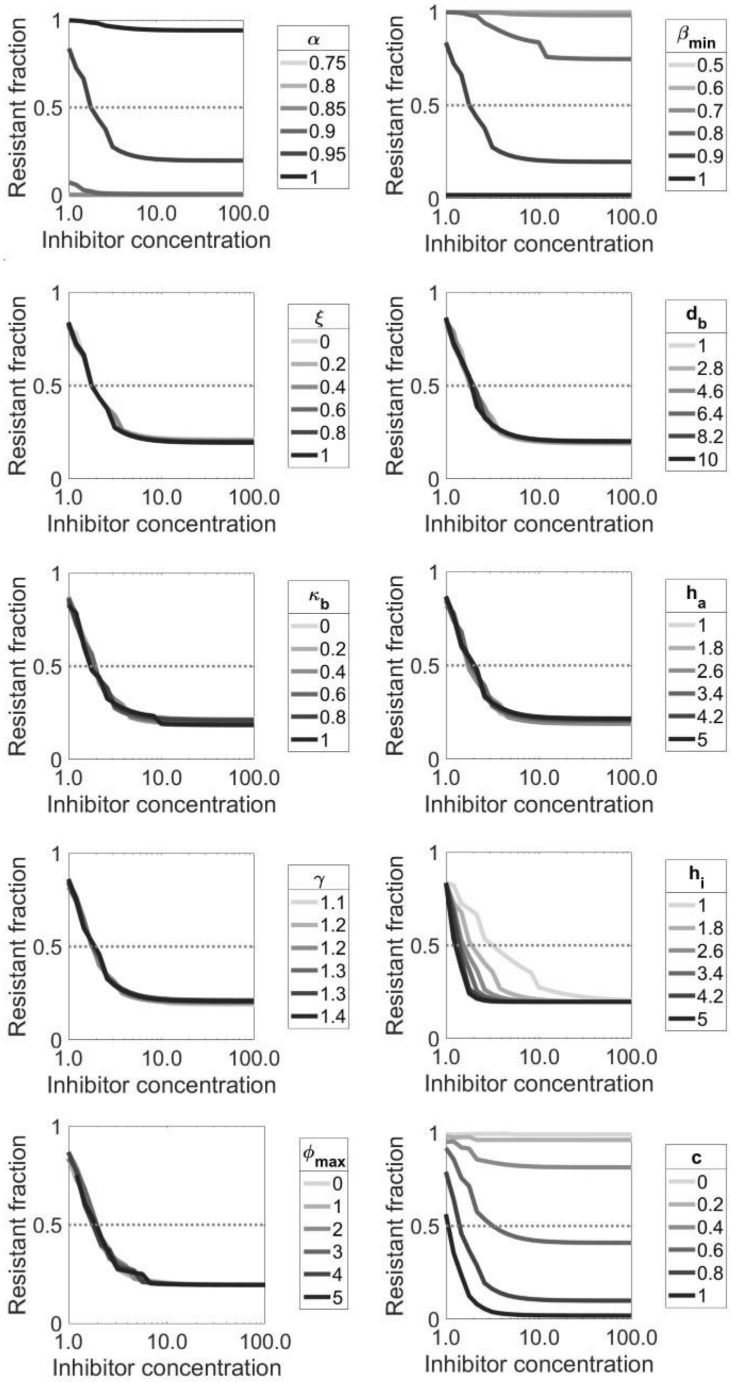

Supplementary Figure S3. Effect of varying each parameter, all else being equal, on the resistant fraction for doses along the minimally effective isobole (see Supplementary Figure S2). With the exception of α, β_{min}, and c, varying parameters has little to no effect on the minimum possible resistant fraction.

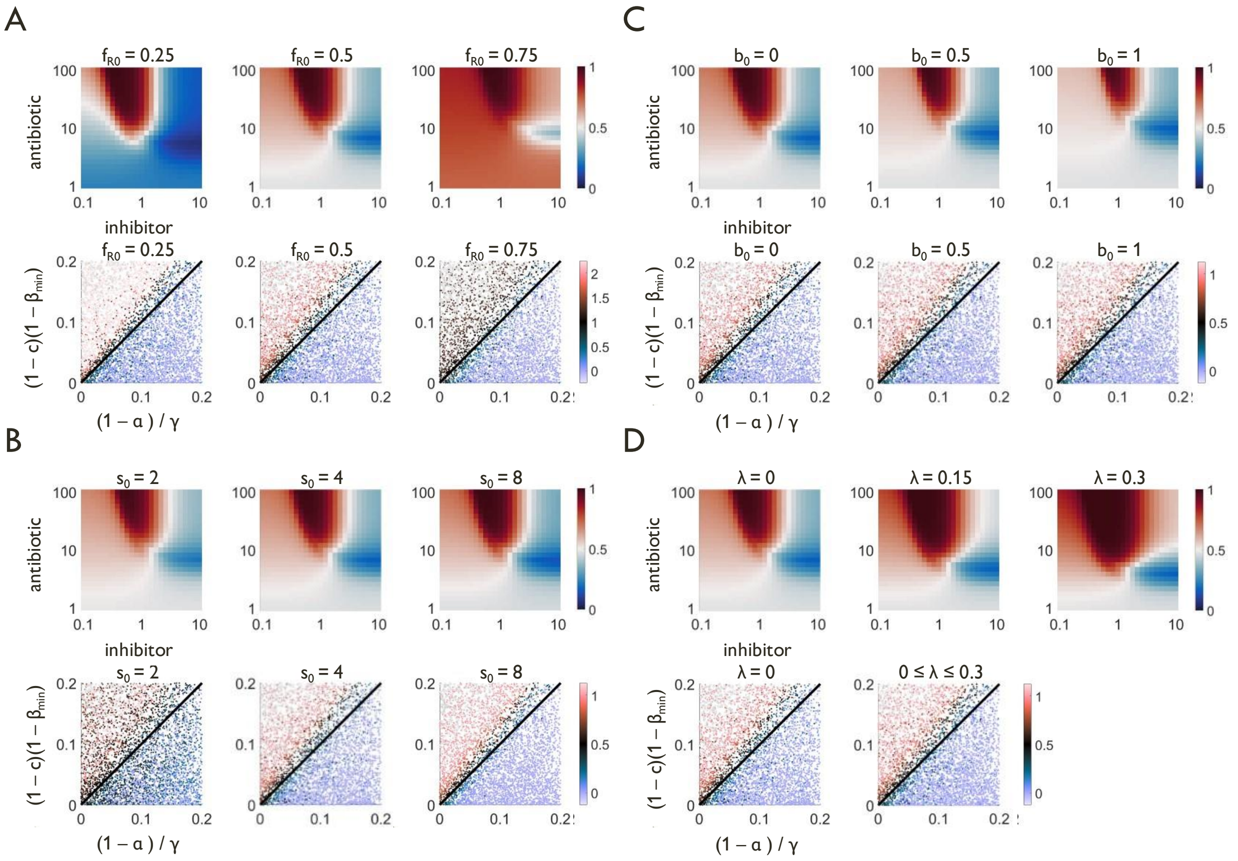

Supplementary Figure S4. Effect of varying initial conditions and growth-lysis association on simulated selection dynamics for the base parameter set (top) and 10K simulated strains with randomized parameters (bottom). Heatmap color indicates final resistant fraction for each antibiotic-inhibitor dose. For panels B-D, dot color for bottom panel indicates minimum achievable resistant fraction for each randomized parameter set. For panel A, the dot color indicates this value divided by the initial resistant fraction. Values below listed from left to right.

A. Direction of selection is insensitive to initial proportion of resistant population. Simulations initialized at $\{n_s(0) = 0.1, n_r(0) = 0.3\}$; $\{n_s(0) = 0.2, n_r(0) = 0.2\}$; and $\{n_s(0) = 0.3, n_r(0) = 0.1\}$. Initial resistant fraction denoted as f_{R0}. Higher initial resistant fractions yield greater final resistance (top), but the direction of selection relative to initial fraction and key parameters is the same for all conditions (bottom).
B. Direction of selection is insensitive to available nutrient. Simulations initialized at $s(0) = 0.2$, $s(0) = 4$, $s(0) = 8$. Increasing nutrient lengthens the timeframe of selection, intensifying selection in either direction (top). For randomized parameter sets, lower nutrient yields more strains with minimal change from initial composition (black dots),

while higher nutrient results in closer association between the criterion and selection outcomes. However, even for lower nutrient, the criterion does not result in misprediction: strains with higher effective private benefit (above the identity line) are not selected against (blue dots) and strains with lower effective private benefit (below the identity line) are not selected for (red dots).

C. Direction of selection is insensitive to initial amount of extracellular Bla. Simulations initialized at $b(0) = 0$, $b(0) = 0.5$, $b(0) = 1$. Initial beta-lactamase shrinks the number of doses at which resistant cells are selected for, by increasing the public good and reducing the time during which resistant cells may have an advantage (top). However, it does not affect the minimum possible resistant fraction (bottom).

D. Direction of selection is insensitive to the incorporation of non-growth-dependent lysis. We modify the form of the lysis equation $l = \gamma \frac{a^{h_a}}{1+a^{h_a}} g$ to $l = \frac{a^{h_a}}{1+a^{h_a}} (\gamma g + \lambda)$ to include the non-growth-dependent lysis term λ. Top simulations were then initialized at $\lambda = 0$, $\lambda = 0.15$, and $\lambda = 0.3$ (ranges selected based on previous work[77]). Including non-growth-dependent lysis increases the number of doses at which resistant cells are selected for. However, it does not affect the minimum possible resistant fraction (bottom).

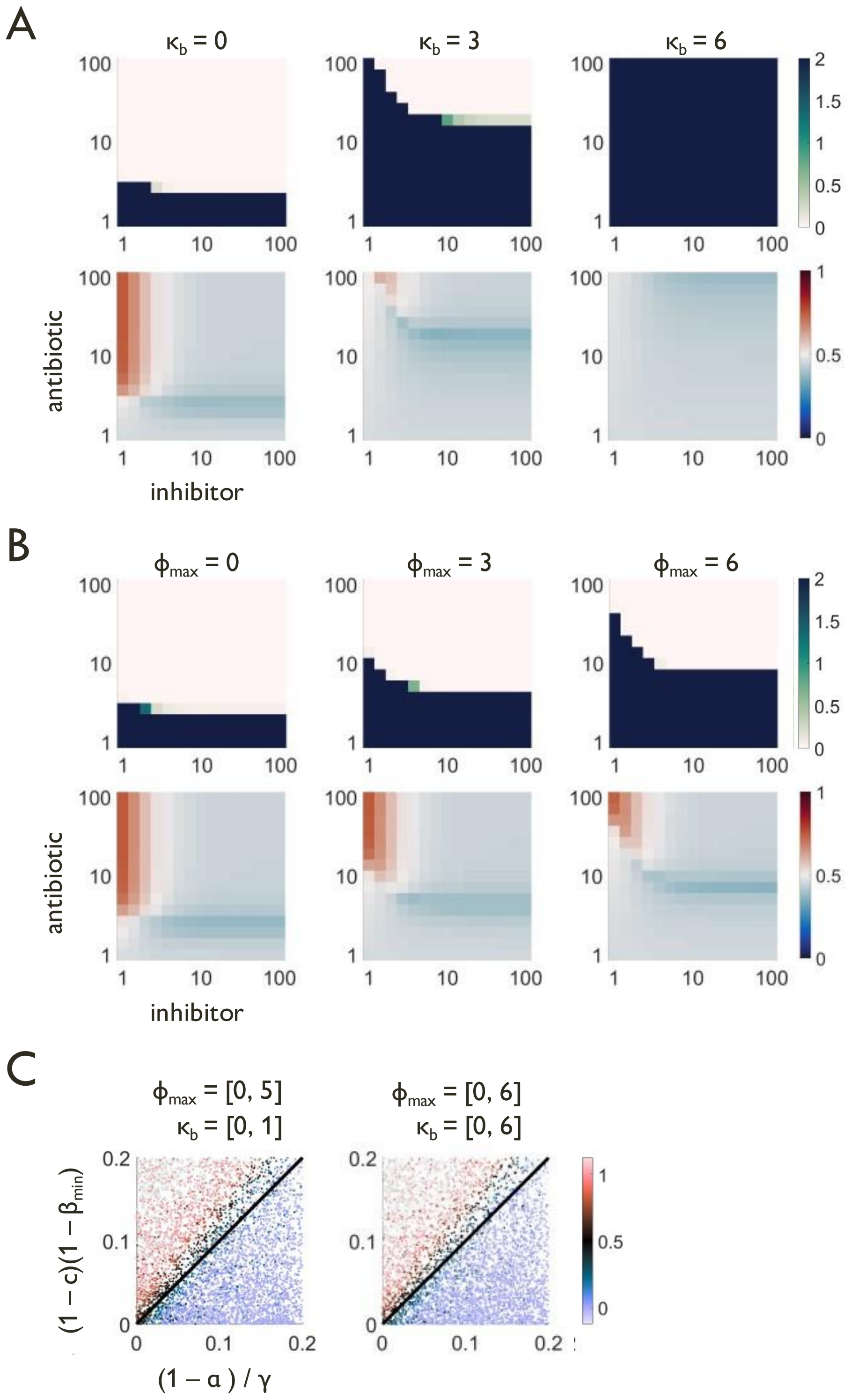

Supplementary Figure S5. Increasing public benefit parameter ranges affects simulated cell survival and selection dynamics, but does not change the minimum achievable resistant fraction for a given landscape.

A. Increasing extracellular Bla-mediated antibiotic degradation increases the range of concentrations that the population can survive (top, heatmap color indicates final cell density for each dose) and at which sensitive cells are selected for (bottom, heatmap color indicates final resistant fraction for each dose). However, it does not affect the magnitude of the minimum achievable resistant fraction (bottom).
B. Increasing intracellular Bla-mediated antibiotic degradation increases the range of concentrations that the population can survive (top, color indicates cell density) and at which sensitive cells are selected for (bottom, color indicates resistant fraction). However, it does not affect the magnitude of the minimum achievable resistant fraction (bottom).
C. Increasing the range of intra- and extracellular Bla-mediated antibiotic degradation parameters when simulating 10,000 strains with random parameter sets does not affect the ability of the criterion to predict the minimum achievable resistant fraction for each strain (dot color). However, an increased range in public benefit parameters does increase the number of simulated strains where no dose was high enough to suppress the population (empty circles).

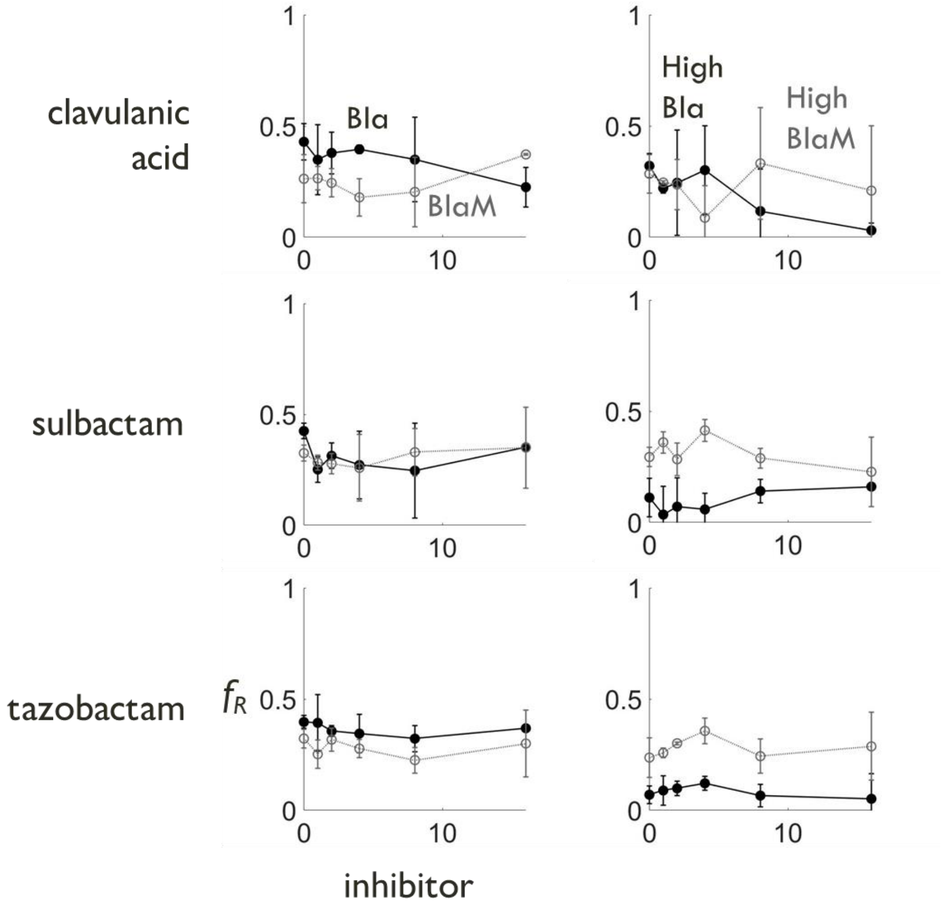

Supplementary Figure S6. Bla (filled black circles) and BlaM (open grey circles) constructs did not exhibit systematic differences in response to the inhibitor alone. These experiments were done in the same manner as those described in Figure 4C (*n*=3 biological replicates), except that no antibiotics were added and the different constructs were exposed to increasing concentrations of different Bla inhibitors.

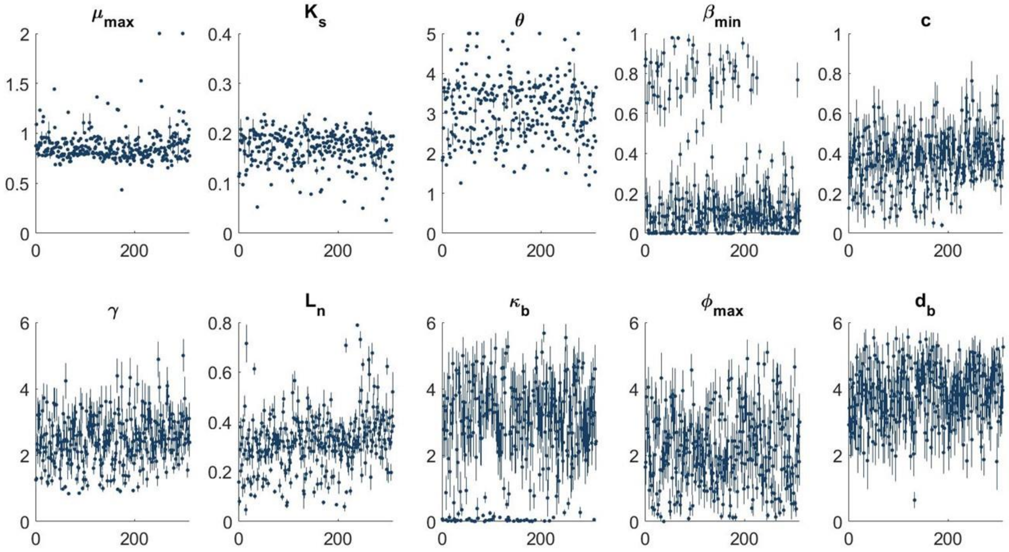

Supplementary Figure S7. Estimated parameters by strain for all 311 isolates. Each dot represents the average of 10 rounds of nonlinear optimization, where initial guesses for each parameter for each round were drawn from a Gaussian distribution (see Supplementary Information, Section 5). Standard error of the mean reported. Note that certain parameters are well constrained by the experimental data, as reflected by the small standard error associated with the estimated value for a parameter. In contrast, estimated values for some other parameters can vary substantially, indicating their 'sloppiness'. Despite the 'sloppiness' of some parameters, the estimated parameters can enable reliable prediction of the experimental data (see Supplementary Figure S8).

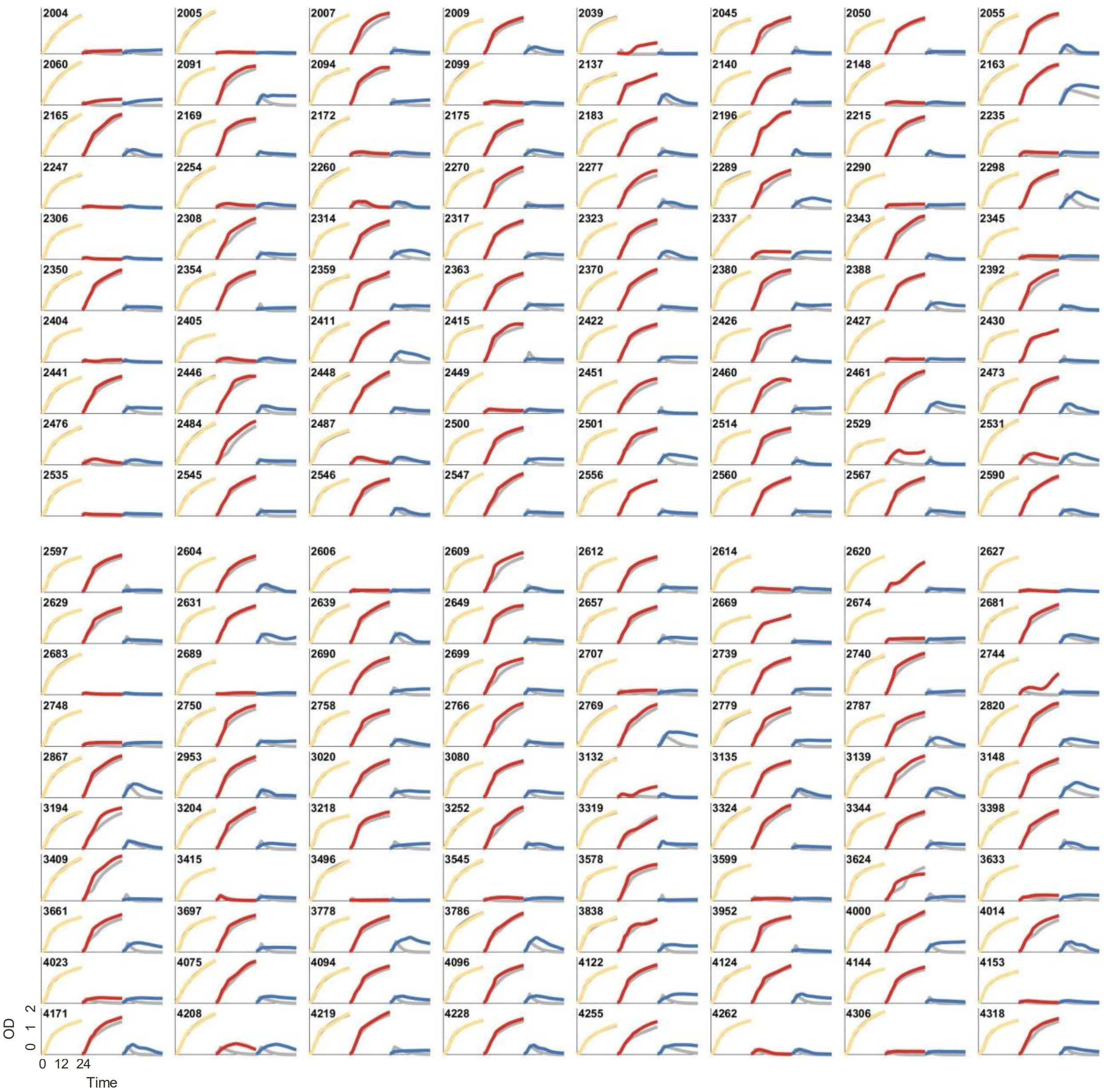
2004 2005 2007 2009 2039 2045 2050 2055
2060 2091 2094 2099 2137 2140 2148 2163
2165 2169 2172 2175 2183 2196 2215 2235
2247 2254 2260 2270 2277 2289 2290 2298
2306 2308 2314 2317 2323 2337 2343 2345
2350 2354 2359 2363 2370 2380 2388 2392
2404 2405 2411 2415 2422 2426 2427 2430
2441 2446 2448 2449 2451 2460 2461 2473
2476 2484 2487 2500 2501 2514 2529 2531
2535 2545 2546 2547 2556 2560 2567 2590
2597 2604 2606 2609 2612 2614 2620 2627
2629 2631 2639 2649 2657 2669 2674 2681
2683 2689 2690 2699 2707 2739 2740 2744
2748 2750 2758 2766 2769 2779 2787 2820
2867 2953 3020 3080 3132 3135 3139 3148
3194 3204 3218 3252 3319 3324 3344 3398
3409 3415 3496 3545 3578 3599 3624 3633
3661 3697 3778 3786 3838 3952 4000 4014
4023 4075 4094 4096 4122 4124 4144 4153
4171 4208 4219 4228 4255 4262 4306 4318
OD
0 1 2
0 12 24
Time

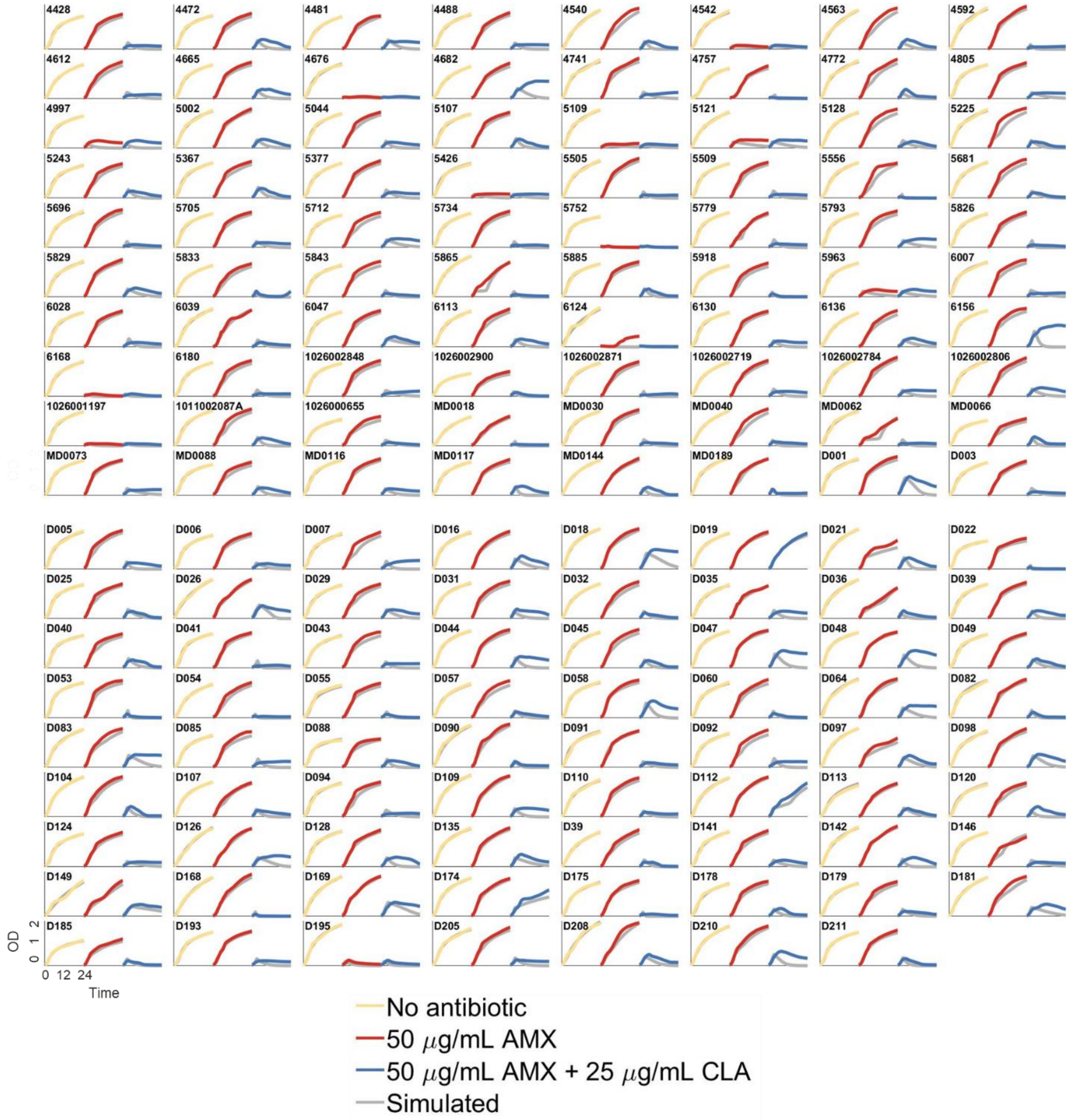

Supplementary Figure S8. Simulated vs experimental data for all 311 isolates, indexed in the same order as in Supplementary Figure 5. All panels have the same x- and y-ranges. Experimental data were collected under 3 conditions: LB only (no antibiotic, beige), LB + 50 μg/ml AMX (red), and LB + 50 μg/ml AMX + 25 μg/ml CLA (blue). OD600 for each condition was measured for 24 hours. Each experimental curve shown above represents the average of all replicates (*n* = 12). The three averaged experimental curves for each condition were used to fit the model parameters (See Supplementary Information). We then used the average value from 10 rounds of estimation for each parameter (Supplementary Figure S7) to predict responses for

each strain for each condition (grey). In general, the fitted parameters enable highly accurate prediction of the experimental data (also see Figure 5C).

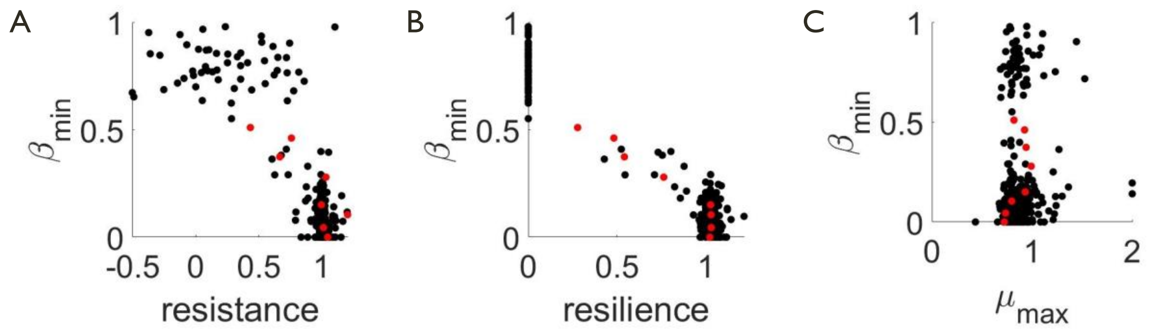

Supplementary Figure S9. Correlation between estimated β_{min} for the library of isolates and other metrics in response to amoxicillin treatment.

(A) Correlation with resistance as defined in Meredith et al.[18]

(B) Correlation with resilience as defined in Meredith et al.[18]

(C) Correlation with estimated μ_{max}.

Isolates chosen for experiments in Figure 5E highlighted in red. They were chosen to be representative of a range of values of β_{min}, resistance, and resilience.

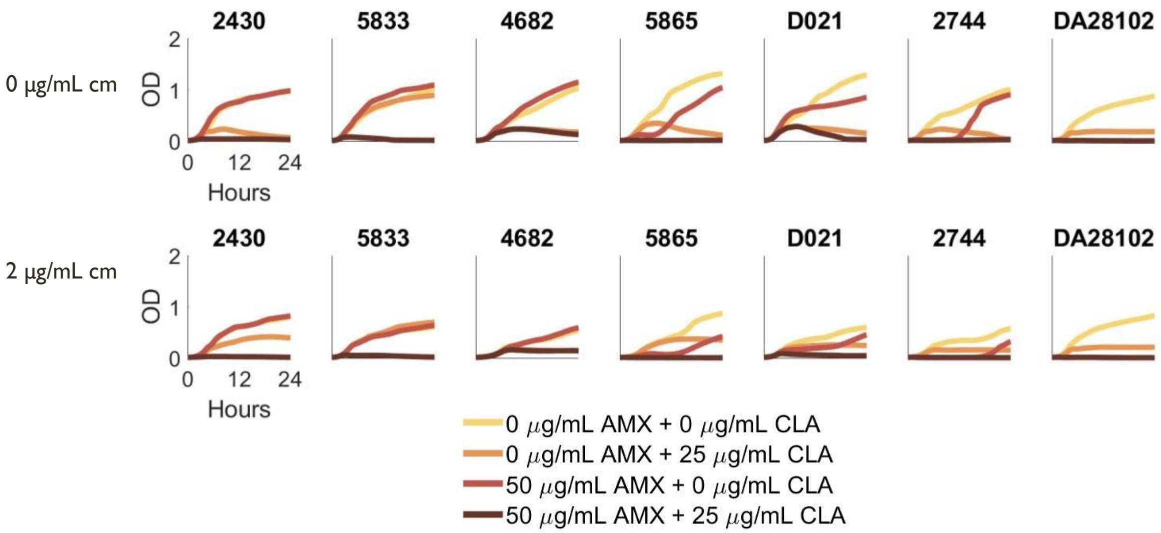

Supplementary Figure S10. Sublethal chloramphenicol affects the speed but not the qualitative shape of responses to amoxicillin and clavulanic acid. In the presence of 2 μg/mL chloramphenicol, the selected clinical isolates grew slightly slower than DA28102, creating the condition where the resistant cells can be selected against in response to β-lactam and Bla inhibitor combination treatment.
