# Supplementary material for: Private benefit of β-lactamase dictates selection dynamics of combination antibiotic treatment
Source: Nat Commun. 2024 Sep 27;15:8337. doi: 10.1038/s41467-024-52711-w (PMC11436977; doi:10.1038/s41467-024-52711-w)
Supplement: Supplementary file 3 — Reporting Summary [file 41467_2024_52711_MOESM3_ESM.pdf]

nature portfolio

Corresponding author(s): Lingchong You

Last updated by author(s): Jul 2, 2024

Reporting Summary

Nature Portfolio wishes to improve the reproducibility of the work that we publish. This form provides structure for consistency and transparency in reporting. For further information on Nature Portfolio policies, see our Editorial Policies and the Editorial Policy Checklist.

Statistics

For all statistical analyses, confirm that the following items are present in the figure legend, table legend, main text, or Methods section.

n/a	Confirmed	
☒	☐	The exact sample size (*n*) for each experimental group/condition, given as a discrete number and unit of measurement
☒	☐	A statement on whether measurements were taken from distinct samples or whether the same sample was measured repeatedly
☒	☐	The statistical test(s) used AND whether they are one- or two-sided<br>*Only common tests should be described solely by name; describe more complex techniques in the Methods section.*
☒	☐	A description of all covariates tested
☒	☐	A description of any assumptions or corrections, such as tests of normality and adjustment for multiple comparisons
☒	☐	A full description of the statistical parameters including central tendency (e.g. means) or other basic estimates (e.g. regression coefficient) AND variation (e.g. standard deviation) or associated estimates of uncertainty (e.g. confidence intervals)
☒	☐	For null hypothesis testing, the test statistic (e.g. *F*, *t*, *r*) with confidence intervals, effect sizes, degrees of freedom and *P* value noted<br>*Give P values as exact values whenever suitable.*
☒	☐	For Bayesian analysis, information on the choice of priors and Markov chain Monte Carlo settings
☒	☐	For hierarchical and complex designs, identification of the appropriate level for tests and full reporting of outcomes
☒	☐	Estimates of effect sizes (e.g. Cohen's *d*, Pearson's *r*), indicating how they were calculated

Our web collection on statistics for biologists contains articles on many of the points above.

Software and code

Policy information about availability of computer code

Data collection	Only instrument-specific commercial software was used to collect data, including Tecan i-control 2.0 and the native software for Keyence BZ-X, Formulatrix MANTIS, and Illumina Mini-Seq instruments.
Data analysis	Data analysis was conducted in MATLAB R2019b, R2023a, R2023b, Python 3, and using Galaxy. Packages include: cmocean, fcrameri, scipy, Trim Sequences, FastQ Joiner, FLASH. Code is deposited in GitHub at https://github.com/youlab/combination-antibiotic-selection.

For manuscripts utilizing custom algorithms or software that are central to the research but not yet described in published literature, software must be made available to editors and reviewers. We strongly encourage code deposition in a community repository (e.g. GitHub). See the Nature Portfolio guidelines for submitting code & software for further information.

Data

Policy information about availability of data

All manuscripts must include a data availability statement. This statement should provide the following information, where applicable:

- Accession codes, unique identifiers, or web links for publicly available datasets
- A description of any restrictions on data availability
- For clinical datasets or third party data, please ensure that the statement adheres to our policy

All experimental and simulation data supporting the findings of this study are available within the manuscript or located in a GitHub repository at https://github.com/youlab/combination-antibiotic-selection.

Research involving human participants, their data, or biological material

Policy information about studies with human participants or human data. See also policy information about sex, gender (identity/presentation), and sexual orientation and race, ethnicity and racism.

Reporting on sex and gender	No human subjects were included in the study.
Reporting on race, ethnicity, or other socially relevant groupings	No human subjects were included in the study.
Population characteristics	No human subjects were included in the study.
Recruitment	No human subjects were included in the study.
Ethics oversight	No human subjects were included in the study.

Note that full information on the approval of the study protocol must also be provided in the manuscript.

Field-specific reporting

Please select the one below that is the best fit for your research. If you are not sure, read the appropriate sections before making your selection.

☒ Life sciences ☐ Behavioural & social sciences ☐ Ecological, evolutionary & environmental sciences

For a reference copy of the document with all sections, see nature.com/documents/nr-reporting-summary-flat.pdf

Life sciences study design

All studies must disclose on these points even when the disclosure is negative.

Sample size	Growth dynamics for dose responses in Figure 1 were conducted in triplicate, except for Figure 1A which was measured with n=5. Population composition was measured in triplicate (biological replicates). Growth dynamics for clinical isolates were measured with n=12 (3 biological x 4 technical replicates). Sample sizes were chosen based on the scale of the project and consistency with similar published studies. No sample size calculation was performed. Our results suggested that the sample sizes chosen were sufficient to evaluate our methodology.
Data exclusions	No data were excluded from the study.
Replication	Experiments were conducted in at least triplicate with similar results, suggesting the robustness of the conclusions.
Randomization	Colonies selected for culture for bacterial isolates were chosen randomly.
Blinding	No group allocation was performed. In this study, knowledge of a subject's identity did not affect experimental conclusions.

Reporting for specific materials, systems and methods

We require information from authors about some types of materials, experimental systems and methods used in many studies. Here, indicate whether each material, system or method listed is relevant to your study. If you are not sure if a list item applies to your research, read the appropriate section before selecting a response.

Materials & experimental systems

n/a	Involved in the study	
☒	☐	Antibodies
☒	☐	Eukaryotic cell lines
☒	☐	Palaeontology and archaeology
☒	☐	Animals and other organisms
☒	☐	Clinical data
☒	☐	Dual use research of concern
☒	☐	Plants

Methods

n/a	Involved in the study	
☒	☐	ChIP-seq
☒	☐	Flow cytometry
☒	☐	MRI-based neuroimaging
